# A population pharmacokinetic model is beneficial in quantifying hair concentrations of ritonavir-boosted atazanavir: a study of HIV-infected Zimbabwean adolescents

**DOI:** 10.1186/s40360-020-00437-y

**Published:** 2020-08-03

**Authors:** Bernard Ngara, Simbarashe Zvada, Tariro Dianah Chawana, Babill Stray-Pedersen, Charles Fungai Brian Nhachi, Simbarashe Rusakaniko

**Affiliations:** 1 Department of Community Medicine, University of Zimbabwe College of Health Sciences, Mazowe Street, Parirenyatwa Complex, P. O Box A178 Avondale, Harare, Zimbabwe; 2 Department of Clinical Pharmacology, Stellenbosch University, Private Bag X1, Matieland, Stellenbosch, 7602 South Africa; 3 Department of Clinical Pharmacology, University of Zimbabwe College of Health Sciences, Mazowe Street, Parirenyatwa Complex, P. O Box A178 Avondale, Harare, Zimbabwe; 4 Institute of Clinical Medicine, Women’s Clinic, Oslo University Hospital, 0027 Oslo, Norway

**Keywords:** Pharmacokinetic modelling, HIV/AIDS, Adolescents, Adherence, Hair, NONMEM

## Abstract

**Background:**

Adolescents experience higher levels of non-adherence to HIV treatment. Drug concentration in hair promises to be reliable for assessing exposure to antiretroviral (ARV) drugs. Pharmacokinetic modelling can explore utility of drug in hair. We aimed at developing and validating a pharmacokinetic model based on atazanavir/ritonavir (ATV/r) in hair and identify factors associated with variabilities in hair accumulation.

**Methods:**

We based the study on secondary data analysis whereby data from a previous study on Zimbabwean adolescents which collected hair samples at enrolment and 3 months follow-up was used in model development. We performed model development in NONMEM (version 7.3) ADVAN 13.

**Results:**

There is 16% / 18% of the respective ATV/r in hair as a ratio of steady-state trough plasma concentrations. At follow-up, we estimated an increase of 30% /42% of respective ATV/r in hair. We associated a unit increase in adherence score with 2% increase in hair concentration both ATV/r. Thinner participants had 54% higher while overweight had 21% lower atazanavir in hair compared to normal weight participants. Adolescents receiving care from fellow siblings had atazanavir in hair at least 54% less compared to other forms of care.

**Conclusion:**

The determinants of increased ATV/r concentrations in hair found in our analysis are monitoring at follow up event, body mass index, and caregiver status. Measuring drug concentration in hair is feasibly accomplished and could be more accurate for monitoring ARV drugs exposure.

## Background

About 36.9 million people were living with Human Immunodeficiency Virus (HIV) worldwide in 2017. Of these, approximately 3 million were children and adolescents under 20 years of age [1]. Zimbabwe has a prevalence of 13.3%, with 1.3 million people living with HIV including 77,000 children and adolescents [2]. Poor adherence to treatment leads to sub-optimal drug exposure limiting treatment efficacy [3, 4]. It has been estimated that between 20 to 50% of adolescents experience adherence-related antiretroviral (ARV) drug treatment failure [5–11].

It is desirable to have a routine assessment of adherence and exposure to ARV drugs available for use by healthcare providers. The methods used for assessing adherence and exposure to ARV drugs include self-reported missed doses, monitoring pharmacy refills and conducting pill counts, use of electronic monitoring devices, measuring ARV concentration in plasma or hair [12–14]. Quantifying ARV drugs in hair provides information of both steady-state pharmacokinetics and long term adherence and has shown to predict well the relationship between drug exposure and treatment outcomes when compared to other approaches [15–21].

Some suggest that hair uptake most external substances or their metabolites from the systemic circulation through the hair bulb blood supply by passive diffusion from blood into growing hair cells at the base of the follicle and then bound in the hair shaft [22–25]. Once the drug accumulates into the growing hair, we can detect it long after elimination from the systematic circulation, unlike in conventional biological samples such as blood and urine [26–30]. The scalp hair fibre grows at an average rate of 0.5 to 1.5 cm per month [31]. Thus the amount of drug in hair is constantly increasing until the next hair cut or when all the drug is removed from the systematic circulation.

In Zimbabwe and other resources limited settings, pharmacokinetic (PK) modelling applied focused primarily on systemic exposure to ARV drugs. It based the models used on data generated by quantifying drugs mostly from single time-point plasma samples [32–35]. Hypothetically hair PK parameters can provide additional information about the patient’s drug exposure overtime, hence the need to determine and apply hair PK for prediction of drug amount in the hair in relation to exposure in plasma.

Using single time-point plasma samples is considered unreliable when assessing drug exposure in populations at risk of non-adherence. Measuring ARV concentrations in hair promises to be more accurate and feasibly accomplished. However, there are very few studies which analysed drug concentration measured in hair using PK approaches. The aim of the work reported in this paper was to develop and validate a population PK model for ATV/r concentrations in hair and explore factors associated with increased or reduced concentrations assuming a direct relationship between ratio in plasma and hair.

## Methods

### Source of data

The study used secondary data from a previously published study conducted in Zimbabwe [15]. They collected the data between January 2015 and May 2016. It comprised 50 adolescents aged between 10 to 18 years and were on ATV/r (300/100 mg) based 2nd line HIV treatment for at least 6 months. They enrolled these participants at a public health hospital in Harare, Zimbabwe, and randomized to either adherence intervention or standard of care arms. They excluded participants if they were on anti-TB treatment, did not prefer home be followed-ups, had viral load < 1000 copies/ml within the previous 2 months, or were on ATV/r as 1st line treatment. The goal of the primary study was to test the impact of a home-based modified directly administered adherence intervention on virologic outcome. They collected questionnaire data, blood samples and hair samples cut closest to the scalp at baseline and at 90 days follow-up. ATV/r in hair were measured using liquid chromatography/mass spectrometry/ mass spectrometry (LC/MS/MS) and the assay range for ATV/r was 0.05–20.0 /0.01–4.0 ng/mg hair, respectively, and a correlation co-efficient of 0.99 for both. Additional details about hair sample preparation and analysis are described in the primary study [15].

### Pharmacokinetic modelling

**Fig. 1:**
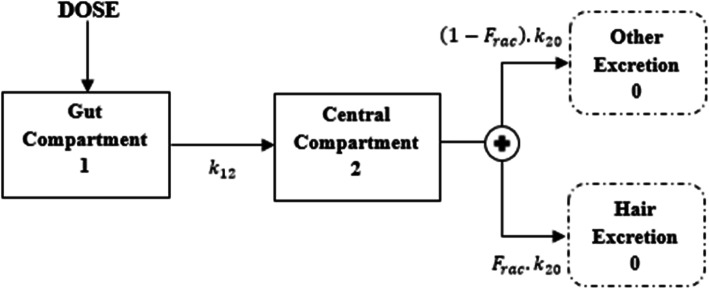
Schematic representation of the structural population PK model used to predict atazanavir and ritonavir concentrations measured in hair

We developed a population PK model to describe ATV/r concentrations in hair. We fixed parameters describing the PK of atazanavir and ritonavir in plasma based on previously published estimates got from studies conducted in almost similar settings [36, 37]. Some of the inter-individual (IIV) and inter-occasion (IOV) variability parameters were estimated while others were fixed in order to improve model fit or stability. We did model development using the first-order conditional estimation method with interaction (FOCE-I) in NONMEM (version 7.3) ADVAN 13 [38]. We schematically presented the structural population PK model applied to both atazanavir and ritonavir concentrations using Fig. 1.

The model describes the concentration of drug in hair at steady-state trough plasma concentrations in the body 24 h after a dose. Given that there were no plasma concentration data, we used a simplified model which estimate ratios of concentrations between hair and plasma. We describe the rate of change of amount of drug between compartments in Fig. 1 using the differential equations:

1 $$ \frac{dA_1}{dt}=-{k}_{12}.{A}_1, $$

2 $$ \frac{dA_2}{dt}={k}_{12}.{A}_1-{k}_{20}.{A}_2, $$

3 $$ \frac{dA_3}{dt}={F}_{rac}\left({k}_{20}\right).{A}_2, $$

4 $$ C=\frac{A_3}{V_h}, $$

Equation 1 describe drug absorption into the plasma circulation i.e. central compartment at a rate (*k*_12_) proportional to the dose amount (*A*_1_); Equation 2 describe input from Equation 1 and total elimination of the drug at rate (${k}_{20}=\frac{CL}{V_c}$, where *CL* and *V*_*c*_ represents clearance and apparent volume of distribution of the bioavailable drug proportional to the amount in the central compartment (*A*_2_); Equation 3 describe a ratio (*F*_*rac*_) of hair concentration relative to steady-state plasma trough concentration. Equation 4 predicts the drug concentration *C* in hair using the ratio of amount of drug in the hair (*A*_3_) and apparent volume of distribution (*V*_*h*_) of the bioavailable drug in the hair.

We tested all covariates in Table 1 during covariate analysis. We selected the optimal covariates relationships through clinical and prior assessment of statistical significance testing using the Stepwise Covariate Model building (SCM) method as implemented in Perl-speaks-NONMEM (PsN). We tested relations on the *F*_*rac*_ parameter only.

**Table 1:** Summary statistics describing data variables of the original study

Variable	Response
Length of hair (cm), median (range)	1 (0.5–1.5)
Hair weight (grams), mean (Standard Deviation; Range)	2.0 (0.15; 1.76–2.28)
Samples Below limit of quantification	
Atazanavir	18 (18)
Ritonavir	12 (12)
Drug regimen: recruitment + follow-up, n (%)	
Tenofovir/Lamivudine/Atazanavir-ritonavir	75 (82)
Abacavir/Didanosine /Atazanavir-ritonavir	6 (7)
Zidovidine/Lamivudine/Atazanavir-ritonavir	6 (7)
Abacavir/Lamivudine/Atazanavir-ritonavir	3 (3)
Tenofovir/ Emtricitabine /Atazanavir-ritonavir	2 (2)
Body mass index-for-age, n (%)	
Normal	25 (54)
Overweight	7 (15)
Thinness	14 (30)
Age (years), mean (Standard Deviation; Range)	15.8 (1.8; 11–18)
Gender, n (%)	
Female	27 (54)
Caregiver, n (%)	
Parent	10 (20)
Grandparent	20 (40)
Sibling	5 (10)
Aunt/uncle	15 (30)
Level of education, n (%)	
Secondary school	39 (89)
Primary school	8 (9)
Dropped	1 (2)
WHO disease progression stage, n (%)	
Early	16 (32)
Late	34 (68)
Adherence by visual inspection of analogue scale, mean (Standard Deviation; Range)	84.2 (18.1; 30–100)
Atazanavir concentration (ng/mg), mean (Standard Deviation; Range)	2.5 (2.0; 0.07–8.65)
Ritonavir concentration (ng/mg), mean (Standard Deviation; Range)	0.5 (0.4; 0.01–1.39)

### Model evaluation

We used the change in objective function value (∆OFV) provided from NONMEM model output at 5% level of significance (i.e. ΔOFV > 3.83, Chi-square 1-degree of freedom) in forward selection process and then at 1% level of significance (i.e. ΔOFV > 6.64, Chi-square 1-degree of freedom) in the backward deletion process, to make discriminations between hierarchical models. We performed bootstrap analysis and 90% confidence intervals on the final covariate models by re-sampling 1000 times in PsN as part of model evaluation numerically. We used graphical assessment of the standard goodness-of-fit plots [39]. Both proportional and additive or combined error models were tested and discriminated by means of change in objective function value (∆OFV).

## Results

### Study participant details

We used 50 participant data in the analysis. The mean (standard deviation) age in years of the study participants was 15.8 (1.8). Fifty-four percent were female. The majority (89%) of these adolescents were attending secondary school, while others were still primary school. Ten percent of the study participants were under the care of fellow siblings below the age of 19 years while others were under the care of parents or relatives. Eighty-two percent of the adolescents were on tenofovir, lamivudine and ATV/r. ATV/r in hair was measured at baseline and 90 days follow-up for every participant and for both drugs, out of the 100 hair samples collected for ATV/r, only 82% / 88%, respectively, were considered for pharmacokinetic modelling, while the remainder 18% / 12% were below the limit of quantification, The median length of hair samples was 1 cm (range 0.5 cm to 1.5 cm). The mean (standard deviation) weight of hair samples was 2.0 g (0.15 g). The mean (standard deviation) drug concentration for atazanavir / ritonavir was 2.5 ng/mg hair (2 ng/mg hair)/0.5 ng/mg hair (0.4 ng/mg hair) respectively. The mean (standard deviation) adherence level of a visual analogue scale of 0 to 100% was 84.1% (18.1%). Further we present details on characteristics of the study population in Table 1.

### Population pharmacokinetic modelling

#### Atazanavir model

We fixed the parameters describing the steady-state population pharmacokinetics of atazanavir in the plasma to 0.44 l per hour for *k*_12_, 10 l per hour for *CL/F* and 63.4 l for *V*_*c*_ [36, 37]. We included body weight as a covariate on *CL/F* and *V*_*c*_ through allometric scaling, fixing the exponents to 0.75 and 1 for *CL/F* and *V*_*c*_ respectively [40]. We initially estimated *V*_*h*_ for atazanavir in hair but later on fixed it to 1 because the model estimates were close to 1 and also to stabilise the final model results. We estimated that ATV concentration measured in hair is approximately16% the amount of atazanavir plasma trough concentration after adjusting for covariate effects. Covariate model results show that participants had ATV concentration 30% less at enrolment than that at follow-up event (*p*-value < 0.0001). A unit increase in self-reported adherence score increased ATV concentration by 2% (p-value = 0.0004). Thinner participants had 54% higher ATV concentration, while overweight participants had 21% lower ATV concentration compared to participants with normal body mass index-for-age (p-value = 0.0165). Participants receiving care from a parent and uncle or aunt had atazanavir in hair 53 and 12% higher respectively, while those receiving care from fellow siblings had atazanavir in hair 54% lower compared to participants receiving care from grandparents (p-value = 0.0406). Based on the change in the OFV value, the most significant covariate was the follow-up occasion (ΔOFV = 47.8, d.f = 1); followed by adherence score (ΔOFV = 18.4, d.f = 1); Body Mass Index-for-age (ΔOFV = 7.5, d.f = 2); and guardian status (ΔOFV = 14.0, d.f = 2), respectively. We present the detailed results in Table 2 and Table 3.

**Table 2:** Effect of covariate inclusion on the OFV for the atazanavir in hair model

Model	OFV	ΔOFV	Cummulative ΔOFV	Cummulative D.F
Baseline	268.6	–	–	–
Baseline+Occassion	220.8	47.8	47.8	1
Baseline+Occasion+Adherence	202.4	18.4	66.2	2
Baseline+Occasion+ Adherence +BMI	194.9	7.5	73.7	4
Baseline+Occasion+ Adherence VAS + BMI + Guardian	180.9	14.0	87.7	7

**Table 3:** Final model parameters describing joint fixed plasma and hair pharmacokinetics of atazanavir

Parameter	Population mean (SE as %)	1000 samples bootstrap medians (90% CI)	Variability (SE as %)	1000 samples bootstrap medians (90% CI)
*k*_12_ (litres hour^− 1^)	0.44 fixed	0.44 fixed	0.45 fixed	0.44 fixed
*CL/F* (litres hour^−1^)	10 fixed	10 fixed	1.04 (99)	0.97 (0.50–1.98)
*V*_*c*_ (litres)	63.4 fixed	63.4 fixed	0.50 fixed	0.50 fixed
*F*_*rac*_	0.16 (16)	0.15 (0.06 to 0.26)		
*V*_*h*_ (litres)	1 fixed	1 fixed		
Occasion (Follow-up)_ *F*_*rac*_	*	*		
Occasion (Enrolment)_ *F*_*rac*_	−0.30 (23)	−0.27 (−0.50 to −0.07)		
Adherence score_ *F*_*rac*_	0.02 (18)	0.015 (0.004 to 0.017)		
Body Mass Index-for-age (Normal)_ *F*_*rac*_	*	*		
Body Mass Index-for-age (Thin)_ *F*_*rac*_	0.54 (22)	0.49 (0.06 to 0.74)		
Body Mass Index-for-age (Overweight)_ *F*_*rac*_	−0.21 (121)	− 0.15 (− 0.26 to − 0.05)		
Guardian (Grandparent)_ *F*_*rac*_	*	*		
Guardian (Parent)_ *F*_*rac*_	0.53 (56)	0.55 (0.03 to 2.58)		
Guardian (Uncle/Aunt)_ *F*_*rac*_	0.12 (177)	0.17 (0.05 to 1.60)		
Guardian (Sibling)_ *F*_*rac*_	−0.54 (35)	−0.60 (− 0.92 to − 0.27)		
*ɛADD*	0.30 (1)	0.29 (0.14 to 0.44)		
*ɛPROP*	0.50 (2)	0.50 (0.39 to 0.61)		
*σ*	1	1		

*k*_12_: Absorption rate constant; CL/F: apparent drug clearance; *V*_*c*_ and *V*_*h*_: apparent volume of distribution in the central and hair compartments, respectively; *F*_*rac*_ amount of drug cleared into the hair as a proportion of the amount of drug in plasma at steady-state troughs; FACTOR_ *F*_*rac*_: effect of covariate on *F*_*rac*_; ɛ_ADD_ and ɛ_PROP_: additive and proportional error terms, respectively; *σ*: residual error; SE: standard error. *: reference group

#### Ritonavir model

We fixed the parameters describing the steady-state population pharmacokinetics of ritonavir in the plasma to 2.31 l per hour for *k*_12_, 12.8 l per hour for *CL/F* and 105 l for *V*_*c*_ [36, 37]. We initially estimated *V*_*h*_ for ritonavir in hair but later on fixed it to 1 because the model estimates were close to 1 and also to stabilise the final model results. We estimated that ritonavir concentration measured in hair is approximately 18% the amount of ritonavir plasma trough concentration after adjusting for covariate effects. Covariate model results show that participants had ritonavir fraction 42% less at enrolment than that at follow-up event (*p*-value = 0.0003). A unit increase in self-reported adherence score increased ritonavir concentrations by 2% (p-value = 0.0245). Based on the change in the OFV value, the most significant covariate was the follow-up occasion (ΔOFV = 14.8, d.f = 1) and then followed by adherence score (ΔOFV = 5.3, d.f = 1). We present the detailed results in Tables 4 and 5.

**Table 4:** Effect of covariate inclusion on the OFV for the ritonavir in hair model

Model	OFV	ΔOFV	Cummulative ΔOFV	Cummulative D.F
Baseline	−61.2	–	–	–
Baseline+Occassion	−76.0	14.8	14.8	1
Baseline+Occasion+Adherence	− 81.3	5.3	20.1	2

**Table 5:** Final model parameters describing joint fixed plasma and hair pharmacokinetics of ritonavir

Parameter	Population mean (***SE*** as %)	1000 samples bootstrap medians (90% CI)	Variability (***SE*** as %)	1000 samples bootstrap medians (90% CI)
*k*_12_ (litres hour^−1^)	2.31 fixed	2.31 fixed	0.45 fixed	0.45 fixed
*CL/F* (litres hour^−1^)	12.8 fixed	12.8 fixed	0.28 (31)	0.28 (0.01 to 0.68)
*V*_*c*_ (litres)	105 fixed	105 fixed	0.50 fixed	0.50 fixed
*F*_*rac*_	0.18 (16)	0.18 (0.14 to 0.21)		
*V*_*h*_ (litres)	1 fixed	1 fixed		
Occasion (Follow-up)_ *F*_*rac*_	*	*		
Occasion (Enrolment)_ *F*_*rac*_	−0.42 (22)	−0.39 (−0.56 to −0.21)		
Adherence score_ *F*_*rac*_	0.02 (47)	0.014 (0.008 to 0.017)		
*ɛADD*	0.34 (95)	0.36 (0.04 to 0.63)		
*ɛPROP*	0.26 (26)	0.24 (0.13 to 0.31)		
*σ*	1	1		

*k*_12_: Absorption rate constant; *CL/F*: apparent drug clearance; *V*_*c*_ and *V*_*h*_: apparent volume of distribution in the central and hair compartments, respectively; *F*_*rac*_: amount of drug cleared into the hair as a proportion of the amount of drug in plasma; FACTOR_ *F*_*rac*_: effect of covariate on *F*_*rac*_; *ɛ*_*ADD*_ and *ɛ*_*PROP*_: additive and proportional error terms, respectively; *σ*: residual error; *SE*: standard error. *: reference category

### Model diagnostics

**Fig. 2:**
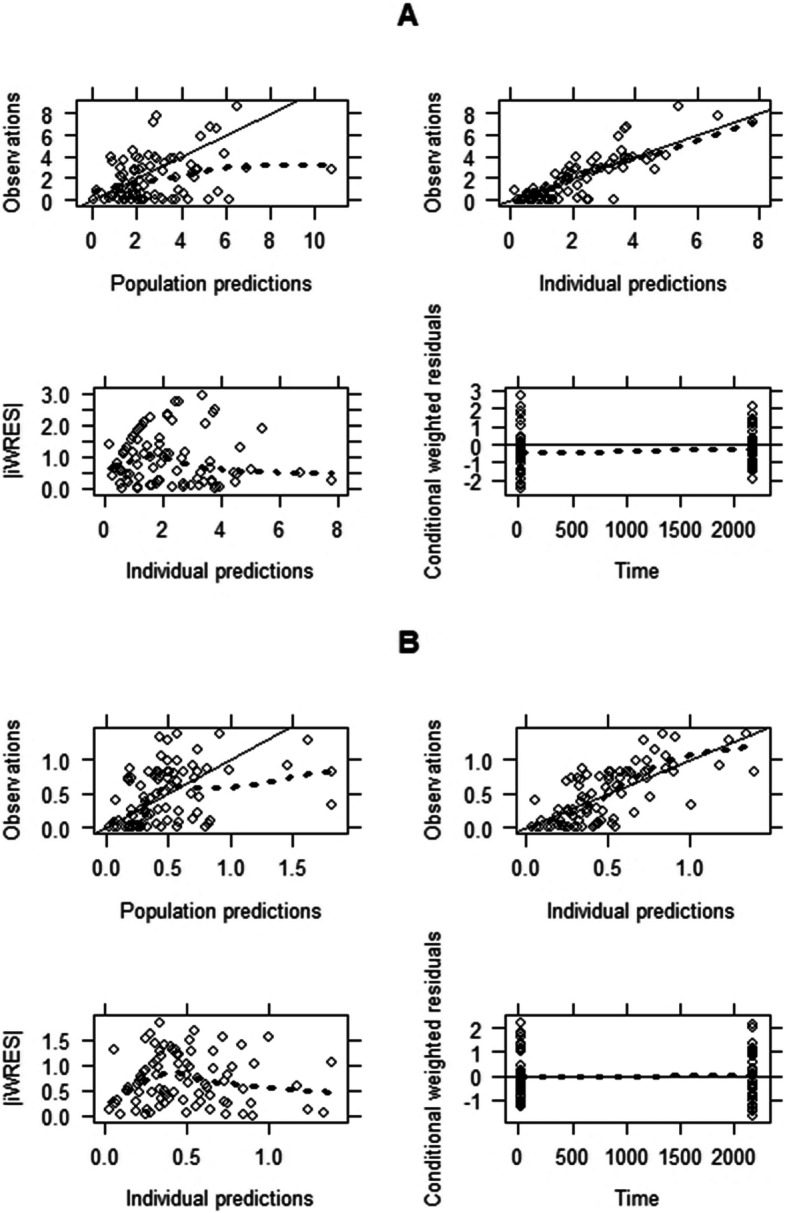
Basic goodness-of-fit plots for the final model for atazanavir 300 mg (**a**) and ritonavir 100 mg (**b**). *Upper left panel:* The observations are plotted versus the population predictions. *Upper right panel:* The observations are plotted against the individual predictions. *Lower left panel:* The individually weighted residuals are plotted versus the individual predictions. *Lower right panel:* The conditional weighted residuals are shown versus time (in hours). The open black circles represents observed data. The bold-dashed line is a locally weighted scatter-plot smoother (LOESS), while the solid line is identity or zero

There was no huge variation between all the estimated final model parameters and those got using 1000 samples bootstrap. All the estimated final model were falling within the 90% confidence intervals. Figure 2 presents the basic goodness-of-fit plots showing the population model predictions versus observations and the residual error plots for both atazanavir and ritonavir final models. The results show low bias, and fairly good precision showing fairly acceptable predictive performance.

## Discussion

This is a breakthrough study to perform joint pharmacokinetic modelling of plasma and hair drug concentrations, determine the relationship between exposure of the drug in hair and that to plasma. Several studies have used drug concentration as a tool for measuring antiretroviral drug exposure in situations where non-adherence to treatment maybe a challenge. However the choice of the multivariate statistical models involving hair concentration as the outcome variable in these studies lacked the dose component which plays a critical role when optimising the relationship between drug exposure and treatment outcomes [5, 15, 17–20]. A non-linear mixed effect PK model has an advantage that includes the dose component. The main purpose of the current model is basically to inform future study design that involve measuring drug concentrations in hair. Later in the discussion, we will present some limitations and recommendations that can improve the power of this method.

Some of the plasma pharmacokinetic parameter we reported while fixing to constant values for the drugs varied from those reported in studies conducted in almost similar settings [36, 37], this could be as a result that some of these values were adjusted by the median body weight observed in our study using allometric scaling. While it is novel to use transit compartment as applied in one of these studies versus the conventional approach (Tlag) to cater for delay in absorption, the disease severity experienced was different in our case due to lower age groups in the previously published study [37]. There is a possibility that certain enzymes and or transporters played a role which physiologically were not possible to incorporate in our model. Hence these two studies were used as a better reference in our study because they were comparable to our study population geographically but had no influence in selection of all parameters describing plasma pharmacokinetics.

The amount of atazanavir concentration determined in hair is approximately 16% of steady-state plasma trough. We estimated an almost similar ratio of 18% for ritonavir as part of model testing. In our conceptual framework, we are interested in finding covariates that affect drug exposure to improve the dosing strategies. We used the SCM in identifying covariates associated with variation in hair drug exposure. The drug that accumulates in hair comes from plasma, therefore one of the major assumption is that the covariates found to have an association with accumulation of drug in hair in our results are a function of an altered plasma PK profile.

ATV/r concentrations increased on follow-up occasion irrespective of study arm which could result from design biases the original study could not eliminate. By being involved in a study, participants are more aware that they are under investigation, hence they adhere more to treatment, increasing hair drug concentrations in both arms. The primary study randomized participants to study arms without blinding, so this could have introduced the biases. High body mass index (BMI)-for-age decreased atazanavir concentrations in hair, while low body mass index-for-age increased atazanavir accumulation in hair. These findings concur with earlier studies in adults [41, 42]. They associated low BMI with high plasma drug concentrations, often resulting in supra-therapeutic drug concentrations, with subsequent drug toxicity, side effects and defaulting treatment. Furthermore, the same literature associates high BMI with low plasma drug concentrations, often resulting in sub-therapeutic drug concentrations and subsequent treatment failure and drug resistance. To the best of our knowledge, our study is the first to show this association in adolescents and using drug concentrations in hair.

We associated receiving care from siblings with lower drug concentrations in hair. Based on current knowledge, this is the 1st study to prove this association using hair samples. Results from a different previous study showed higher self-reported adherence in children and youth who stayed with their parents and grandparents, than those who stayed with siblings [43]. Siblings of HIV-infected children and youth are often immature themselves and still coming to terms with burdens associated with child-headed families, orphan-hood and poverty. The needs of HIV-infected children come in as an extra burden that the siblings may not manage the pressure that comes with the burden, leading to missed doses and hospital visits, and subsequent treatment failure in the HIV-infected adolescents.

A major limitation of the modelling approach applied in the article is we had a small sample size (*n* = 50) and that we got only two times-points of drug concentration data from each participant. An additional number of participants coupled with having several or segmental measurement of drug concentration from the hair and additionally measuring drug concentration in plasma will improve the power of the modelling framework that used in this paper. Using prior estimates on the plasma PK model could have led to an underestimation of steady state trough concentration because of unavailability of adherence data, however including prior estimates in the form of both fixed and random effects on the plasma PK model could have reduced the bias. Most of the study participants (82%) were on a uniform drug combination, however the unavailability of data on non-HIV/AIDS linked co-therapies limited investigations of the drug-drug interactions which is also very critical to test during PK analysis. Also, apart from the low sample size, the unavailability of data about how the participants cosmetically treated their hair before it was sampled for the study can possible explain why some of the model parameters were reported with notable very high residual standard errors, hence limiting the accuracy of the model estimates.

## Conclusion

We have showed some work which can complement the efforts being taken by other scientists to establish the use of measuring drug concentration in hair at HIV/AIDS points of care. Most important determinants of increased concentrations in hair were monitoring at follow up event, BMI-for-age and caregiver. Measuring ARV concentrations in hair promises to be more accurate and feasibly accomplished. It is crucial to perform follow-up work which involves establishing the relationship between hair drug concentrations and a measure of treatment response such as viral loads. Comparing the predictive accuracy for exposure-response models when exposure of interest is plasma or hair drug concentrations is necessary to perform.

## Data Availability

The primary study did not publish the data to the public, however the data can be available upon request and approval from the principal investigators of the primary study.

## Abbreviations

ARV: Antiretroviral
ATV/r: Atazanavir/ritonavir
NONMEM: Nonlinear mixed effect modelling
HIV: Human immunodeficiency virus
AIDS: Acquired immunodeficiency syndrome
BMI: Body mass index
PK: Pharmacokinetic
IIV: Inter-individual variability
IOV: Inter-occasion variability
FOCE-I: First-order conditional estimation method with interaction
PsN: Perl-speaks-NONMEM
SCM: Stepwise covariate model building
∆OFV: Change in objective function value
Ng/mg: Nanogram per milligram
*k*_12_: Absorption rate constant
CL/F: Apparent drug clearance
*V*_*c*_: Apparent volume of distribution in the central compartments
*V*_*h*_: Apparent volume of distribution in the hair compartments
*F*_*rac*_: Amount of drug cleared into the hair as a proportion of the amount of drug in plasma at steady-state troughs
FACTOR_ *F*_*rac*_: effect of covariate on *F*_*rac*_
ɛ_ADD_: Additive error term
ɛ_PROP_: Proportional error term
Ω: Residual error
SE: Standard error
*P*-value: Probability value
CI: Confidence interval
